# Tumour-derived extracellular vesicles in blood of metastatic cancer patients associate with overall survival

**DOI:** 10.1038/s41416-019-0726-9

**Published:** 2020-01-15

**Authors:** Afroditi Nanou, M. Craig Miller, Leonie L. Zeune, Sanne de Wit, Cornelis J. A. Punt, Harry J. M. Groen, Daniel F. Hayes, Johann S. de Bono, Leon W. M. M. Terstappen

**Affiliations:** 1 Department of Medical Cell BioPhysics, Faculty of Science and Technology, University of Twente, Enschede, The Netherlands; 2 ClarifyDx Consulting, Quakertown, PA USA; 3 Department of Medical Oncology, Academic Medical Center, University of Amsterdam, Amsterdam, The Netherlands; 4 Department of Pulmonary Diseases, University of Groningen and University Medical Centre of Groningen, Groningen, The Netherlands; 5 Department of Internal Medicine, University of Michigan Rogel Cancer Center, Ann Arbor, MI USA; 6 Prostate Cancer Targeted Therapy Group, The Royal Marsden NHS Foundation Trust, London, UK; 7 Division of Clinical Studies, The Institute of Cancer Research, Royal Marsden Hospital, London, UK

**Keywords:** Tumour biomarkers, Fluorescence imaging, Prognostic markers, Metastasis

## Abstract

**Background:**

Circulating tumour cells (CTCs) in blood associate with overall survival (OS) of cancer patients, but they are detected in extremely low numbers. Large tumour-derived extracellular vesicles (tdEVs) in castration-resistant prostate cancer (CRPC) patients are present at around 20 times higher frequencies than CTCs and have equivalent prognostic power. In this study, we explored the presence of tdEVs in other cancers and their association with OS.

**Methods:**

The open-source ACCEPT software was used to automatically enumerate tdEVs in digitally stored CellSearch® images obtained from previously reported CTC studies evaluating OS in 190 CRPC, 450 metastatic colorectal cancer (mCRC), 179 metastatic breast cancer (MBC) and 137 non-small cell lung cancer (NSCLC) patients before the initiation of a new treatment.

**Results:**

Presence of unfavourable CTCs and tdEVs is predictive of OS, with respective hazard ratios (HRs) of 2.4 and 2.2 in CRPC, 2.7 and 2.2 in MBC, 2.3 and 1.9 in mCRC and 2.0 and 2.4 in NSCLC, respectively.

**Conclusions:**

tdEVs have equivalent prognostic value as CTCs in the investigated metastatic cancers. CRPC, mCRC, and MBC (but not NSCLC) patients with favourable CTC counts can be further prognostically stratified using tdEVs. Our data suggest that tdEVs could be used in clinical decision-making.

## Background

Characterisation of a patient’s tumour is frequently assessed on the primary tumour. However, it is well recognised that the phenotype of a tumour is highly heterogeneous; hence, a biopsy in a selective area is very restrictive in representing the whole tumour.^1,2^ Moreover, the lesions in metastatic sites further complicate the tumour pattern as they can have substantially different features than the primary tumour.^3–5^ In addition, tumours evolve over time and treatments while developing drug-resistance mechanisms.^6^ From these observations emerge the importance of a biopsy that could provide clinicians with real-time data to facilitate treatment decision-making. In theory, subsequent collections of solid biopsies from multiple sites could be a solution; however, in practice, not all sites are accessible by surgery and such a procedure is highly invasive leading to patient discomfort and health complications. On the other hand, liquid biopsies require a minimally invasive biofluid sampling; consequently, they can be performed in short time intervals providing clinicians with a real-time snapshot of the disease.^7^ Importantly, the detected tumour material in biofluids, namely circulating tumour cells (CTCs), tumour-derived extracellular vesicles (tdEVs) and circulating tumour DNA (ctDNA), is clinically relevant and can better reflect the characteristics of the metastatic sites.^8^

Specifically, the field of tdEVs has gained a lot of attention during the past few years, mainly because of their increased prevalence and promise as potential biomarkers to aid in the disease management of cancer patients.^9–15^ The reported size range of tdEVs varies between 30 and 10,000 nm, with the diameter of exosomes being 30–300 nm, of microvesicles <1000 nm and of large oncosomes between 1000 and 10,000 nm.^16–18^ All different tdEV subclasses have been reported to play various roles directly related to the disease progression and metastatic processes.^19–25^ However, the isolation, enumeration, differentiation and molecular profiling of pure tdEVs from the blood of cancer patients is challenging because of all the contaminants present, including proteins, protein aggregates, free nucleic acids (RNA and DNA), platelets and EVs of different cell origins.^26,27^ Hence, enrichment and/or depletion techniques are necessary for the specific isolation of tdEVs and their downstream characterisation.

Previously, our group reported the presence of EpCAM+, CK+, DNA−, CD45− “tumour microparticles” and “CTC fragments” that were considered by-products of cancer apoptosis.^28,29^ These particles have relatively large sizes (>1 μm) and can be isolated together with CTCs from 7.5 mL of peripheral blood after immunomagnetic selection targeting the epithelial cell adhesion molecule (EpCAM) expressed on their surface membrane. We used the term tumour-derived EVs (tdEVs) to describe these objects. The isolation and labelling of the EpCAM enriched sample is performed using the Food and Drug Administration (FDA)-cleared CellSearch® system. For the automated enumeration of tdEVs, the open-source ACCEPT software (http://github.com/LeonieZ/ACCEPT) was used. ACCEPT segments all detected objects in the fluorescence images and measures 10 parameters in each fluorescence channel regarding their morphology and fluorescence intensity. By defining and applying linear gates, different classes of objects (leukocytes, CTCs, tdEVs) found in the fluorescence CellSearch images can be enumerated in an automated manner. Following that approach, we previously reported that these large tdEVs are present in the blood of castration-resistant prostate cancer (CRPC) patients in approximately 20 times higher frequencies compared to CTCs and have a significant association with poor prognosis.^14^ Here we explore whether the presence of EpCAM+, CK+, DNA−, CD45− tdEVs in metastatic breast cancer (MBC), metastatic colorectal cancer (mCRC), and non-small cell lung cancer (NSCLC) patients is associated with overall survival (OS) and we determine whether or not tdEVs can further improve prognostication of cancer patients.

## Methods

### Patient samples

One hundred and ninety CRPC (IMMC38 clinical trial, NCT00133900),^30^ 450 mCRC (CAIRO II clinical trial, NCT00208546),^31,32^ 179 MBC (IMMC01 clinical trial),^33^ and 137 NSCLC patient samples,^34–36^ along with 93 healthy control samples (IMMC06 clinical trial, NCT00133913),^37^ were included. The included samples corresponded to patients before the initiation of a new treatment. Patient characteristics are provided in Table 1. All individuals provided written informed consent prior to participation in the trial protocols approved by institutional review boards at the participating centres of the studies.^31–38^

**Table 1:** Patient characteristics.

Metastatic cancer patients	CRPC^a^	MBC	mCRC	NSCLC
Number of patients	190	179	450	137
Age (years)				
Median (range)	70 (49–92)	59 (27–86)	63 (27–83)	65 (29–83)
Gender				
Male	190 (100%)	0 (0%)	271 (60%)	74 (54%)
Female	0 (0%)	179 (100%)	179 (40%)	63 (46%)
Unknown	0 (0%)	0 (0%)	0 (0%)	0 (0%)
ECOG performance status				
0	87 (46%)	83 (46%)	286 (64%)	78 (57%)
1	80 (42%)	73 (41%)	153 (34%)	49 (36%)
2	17 (9%)	17 (10%)	2 (0%)	5 (4%)
3	0 (0%)	1 (1%)		
4	0 (0%)	0 (0%)		
Unknown	6 (3%)	5 (3%)	9 (2%)	5 (4%)
Line of therapy				
1	132 (70%)	75 (42%)	450 (100%)	36 (26%)
2	29 (15%)	27 (15%)		21 (15%)
3+	29 (15%)	75 (42%)		80 (59%)
Unknown		2 (1%)		
Therapy type started after blood draw				
Chemotherapy	11 (6%)	75 (42%)		32 (23%)
Hormone		45 (25%)		
Molecular		8 (4%)		20 (15%)
Immunotherapy				80 (58%)
Other		1 (1%)		
Chemotherapy/hormone	126 (66%)	11 (6%)		
Chemotherapy/molecular		23 (13%)	450 (100%)	
Chemotherapy/other	7 (4%)	1 (1%)		
Hormone/molecular		8 (4%)		
Chemotherapy/molecular/hormone		2 (1%)		
Chemotherapy/hormone/other	45 (24%)			
Unknown	1 (1%)	5 (3%)		5 (4%)
Follow-up time (in months), median (min–max)				
Alive	30.4 (1.9–39.0)	20.6 (1.3–48.8)	20.2 (0.0–34.0)	8.8 (0.7–30.1)
Dead	11.6 (0.7–39.3)	9.9 (0.4–31.7)	12.2 (0.4–32.3)	4.6 (0.7–25.3)
Status at last follow-up				
Alive	53 (28%)	77 (43%)	194 (43%)	68 (50%)
Dead	137 (72%)	102 (57%)	256 (57%)	69 (50%)

^a^In case of CRPC, the line of therapy refers to the line of cytotoxic therapy

### Isolation and identification of CTCs and tdEVs

Digitally stored CellSearch® (Menarini Silicon Biosystems, Huntingdon Valley, PA, USA) image files from the abovementioned CTC studies were re-analysed. Briefly, CTCs and tdEVs were immunomagnetically isolated from 7.5 mL of peripheral blood collected in Cell Save tubes using the CellSearch system. The EpCAM-enriched cells were stained with the nucleic acid dye 4,6-diamidino-2-phenylindole (DAPI) and the staining reagent of the CTC kit including mouse monoclonal antibodies against CD45 (clone HI30) conjugated to allophycocyanin (APC) and mouse monoclonal antibodies against cytokeratins (CKs) 8, 18 and 19 (clones C11 and A53-B/A2) conjugated to phycoerythrin (PE). In case of CRPC, MBC and mCRC, no extra markers were used in the fluorescein isothiocyanate (FITC) and peridinin chlorophyll protein (PerCP) channels. In case of NSCLC, the EpCAM-enriched cells had been additionally labelled with mouse monoclonal antibodies against CD16 (clone 3G8) conjugated to PerCP (Marker 2) and in some of the cases with the wheat germ agglutinin conjugated to Alexa 488 (Marker 1) or with mouse monoclonal antibodies against CKs 1–8, 10, 14, 15, 16, 19, 20 (clones LP5K, Ks20.10 and AE1/AE4) conjugated to FITC (Marker 1) to address research points of previously reported studies. The subsets of patient samples with additional Marker 1 or/and 2 labelling can be found in Supplementary Table S1. The immunofluorescently stained suspension was placed in a cartridge contained within a Magnest® as previously described.^37^ The image acquisition was performed on the CellSpotter^TM^ Analyzer for the older IMMC01 study (MBC) and the healthy donors included in the IMMC06 study. The image acquisition of the IMMC38 (CRPC), CAIRO II (mCRC) and NSCLC studies was performed on the CellTracks® Analyzer II^TM^. Both systems are semi-automated fluorescence microscopes equipped with computer-controlled *X*, *Y*, *Z* stages, a NA 0.45 ×10 objective, a Mercury Arc lamp, a 12-bit CCD camera and filter cubes for DAPI, PE, APC and FITC. Typically, 175 images per channel are taken to cover the entire surface of the cartridge.^39^

### CTC counts and automated enumeration of tdEVs with ACCEPT

To obtain accurate counts of CTCs and tdEVs, the manual CTC counts were extracted from the CellTracks Analyzer II. For tdEV enumeration, the digitally stored fluorescence image files were re-analysed with the open-source ACCEPT software v1.1 (http://github.com/LeonieZ/ACCEPT) using the “Full Detection” function. After that analysis, 10 morphological and fluorescence signal intensity measurements, for each object found in the images, are extracted per channel. These measurements can be used to design linear gates to identify different classes of objects in the images.^14,34^ The definition of tdEVs in case of CRPC, MBC, mCRC was EpCAM+, CK+, DAPI−, CD45− particles of a diameter <14 μm. The background PE fluorescence in the images acquired on the CellSpotter Analyzer was higher compared to the CellTracks Analyzer II, resulting in the use of a different tdEV gate for each platform. The applied tdEV gate for images acquired on CellTracks Analyzer II was: Mean Intensity CD45 ≤5, Mean Intensity DNA ≤5, Mean Intensity CK >60, Mean Intensity Marker 1 ≤5, Mean Intensity Marker 2 ≤5, Max Intensity CK >90, Size CK ≤150 μm^2^, Perimeter CK >5 pixels, Eccentricity CK ≤0.8, and Perimeter to Area CK ≤1.^14^ For the images acquired on the CellSpotter, the setting of Standard Deviation for CK >40 was implemented in the tdEV gate instead of the CK Max Intensity. In case of NSCLC, tdEVs should be additionally CD16− (an already included criterion in the aforementioned tdEV gate as Mean Intensity Marker 2 ≤5). However, tdEVs could be either positive or negative for Marker 1 (wheat germ agglutinin or CKs 1–8, 10, 14, 15, 16, 19, 20). In order to be able to compare among different NSCLC patient samples, we did not include any requirements for the Mean Intensity Marker 1.

### Statistical analysis

Statistical analysis was performed using SPSS 23.0 (SPSS Inc., Chicago, IL, USA). For each cancer type, a two-tailed Spearman’s Rho test was performed to evaluate the relation between the CTC and tdEV counts. The non-parametric Wilcoxon Signed Ranks test was used to test the equality of the distributions for the matched CTC and tdEV counts within each cancer type. The non-parametric Mann–Whitney *U* test was used to test the equality of the distributions for the CTC and tdEV counts in the healthy donors compared to each patient cohort as well as all patients together. OS for each patient was defined as the elapsed time in months between the baseline blood draw date and the date of death or the date of last follow-up. Patients alive at the end of the study or lost during the follow-up period were censored. Median OS was estimated by Kaplan–Meier (KM) survival curves and survival curves for favourable and unfavourable groups based on CTC and/or tdEV counts were compared using the non-parametric log-rank test. Cox proportional hazards regression analysis was used to determine the univariable hazard ratios (HRs) for OS with 95% confidence intervals (CIs). A final multivariable Cox model for each cancer type was fit including the significant variables from the univariable Cox proportional hazards regression analysis. Owing to correlation between some of the included variables, the final model was selected using forward stepwise elimination (*p*_in_ = 0.05 and *p*_out_ = 0.10). The open-source web application Cutoff Finder (http://molpath.charite.de/cutoff) was used to calculate the HRs for OS with 95% CIs over a wide range of cut-off values for the CTC and tdEV counts for the full data sets as well as for tdEV counts in the subset of patients with favourable CTC counts. The optimal cut-off values of tdEVs for patients with favourable CTCs were defined as the points with the most significant split (log-rank test). Cutoff Finder uses the R code to provide optimisation and visualisation tools for cut-off determination.^40^ Receiver operating characteristic (ROC) curves were used for each cancer type to assess the performance of CTCs and tdEVs in classifying patients based on shorter than median OS or death.

## Results

### ACCEPT display of CTCs and tdEVs

**Fig. 1:**
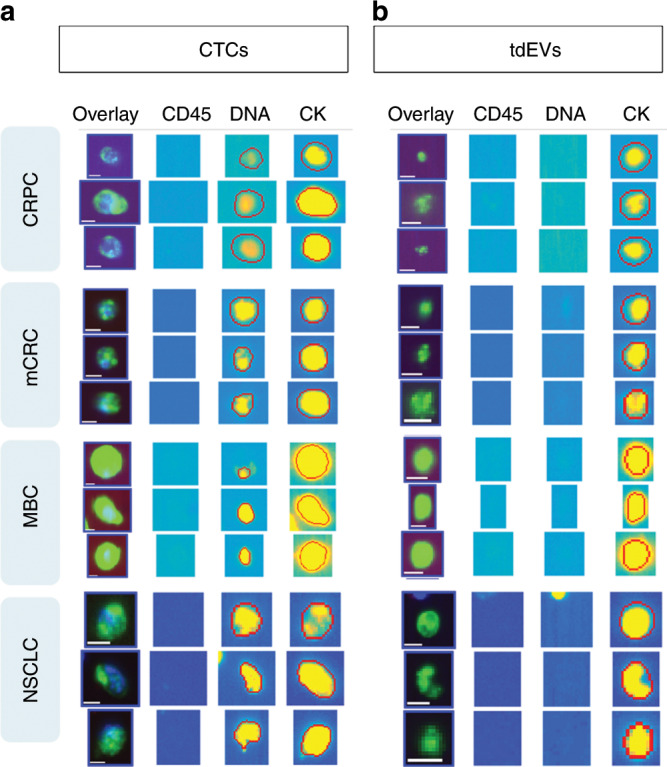
Examples of objects manually classified as CTCs by individual users (**a**) or automatically classified as tdEVs using the open-source ACCEPT software (**b**) on images obtained from CRPC, MBC, mCRC and NSCLC cancer patients. The red contours around the objects indicate the contours as detected by the ACCEPT image analysis algorithm. All objects were isolated from 7.5 mL of blood using the CellSearch system. Scale bars indicate 6.4 μm.

All objects found in the fluorescence images are visualised using the quantitative display of the ACCEPT toolbox. Figure 1 shows examples of manually scored CTCs and automatically scored tdEVs found in the CellSearch images.

### CTC and tdEV frequencies in CRPC, MBC, mCRC and NSCLC

**Fig. 2:**
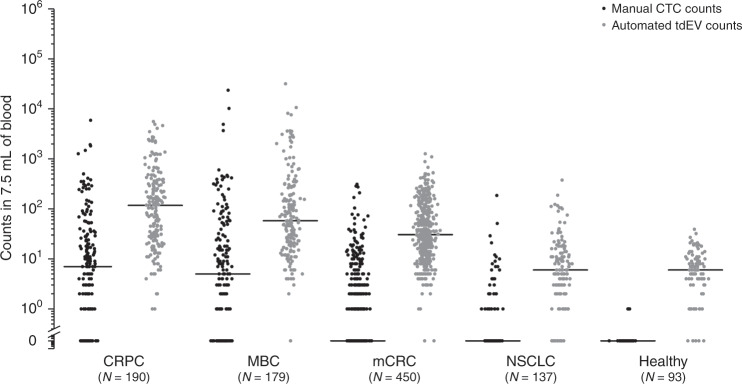
Dot plots of manual CTC counts (in black) and automated tdEV counts (in grey) in the blood of individuals with different cancer types. Horizontal black lines indicate median values. tdEV counts are significantly higher compared to the respective CTC counts in CRPC, MBC, mCRC and NSCLC cancer patients (*p* < 0.05 for all 4 comparisons, Wilcoxon Signed Ranks test). CTC and tdEV counts of 93 healthy donors were used as a reference.

The frequencies of EpCAM+ CTCs and tdEVs identified in blood samples obtained from CRPC, MBC, mCRC and NSCLC patients are provided in Fig. 2. Samples from 93 healthy donors were included as a reference. The CTC distribution of healthy donors was highly significantly different compared to the respective distribution of CRPC, MBC and NSCLC (*p* < 0.001, Mann–Whitney *U* test) but not mCRC (*p* > 0.05, Mann–Whitney *U* test). In case of tdEVs, the distribution of healthy donors was highly significantly different compared to the respective distribution of CRPC, MBC and mCRC, but not NSCLC. Comparison of the pooled patient CTC and tdEV data set to the reference data set of healthy donors resulted in a highly significantly different distribution of only tdEVs. Notably, the median and average tdEV counts are an order of magnitude higher compared to the respective CTC counts (*p* < 0.01, Wilcoxon Signed Ranks test), with 96.4% of all patients (186/190 CRPC, 169/179 MBC, 448/450 mCRC and 119/137 NSCLC patients) having higher tdEV counts. CRPC patients had the highest median CTC and tdEV counts, followed by MBC, mCRC and NSCLC patients. CTC and tdEV counts were significantly correlated in all cancer types, as illustrated in Supplementary Fig. S1. The correlation coefficient between CTC and tdEV counts in these cancer types were tested using Spearman’s Rho test and was found to be 0.87 for CRPC (*p* < 0.001), 0.70 for MBC and mCRC (*p* < 0.001) and 0.44 for NSCLC cancer (*p* < 0.001).

Owing to skewed distribution, we chose to use the median value plus 2 standard deviations (SD) of tdEV counts detected in the 93 healthy donors as a normal reference range. This resulted in a normal range of 0–20 tdEVs per 7.5 mL of blood.

### Association of CTCs and tdEVs with OS in cancer patients

**Fig. 3:**
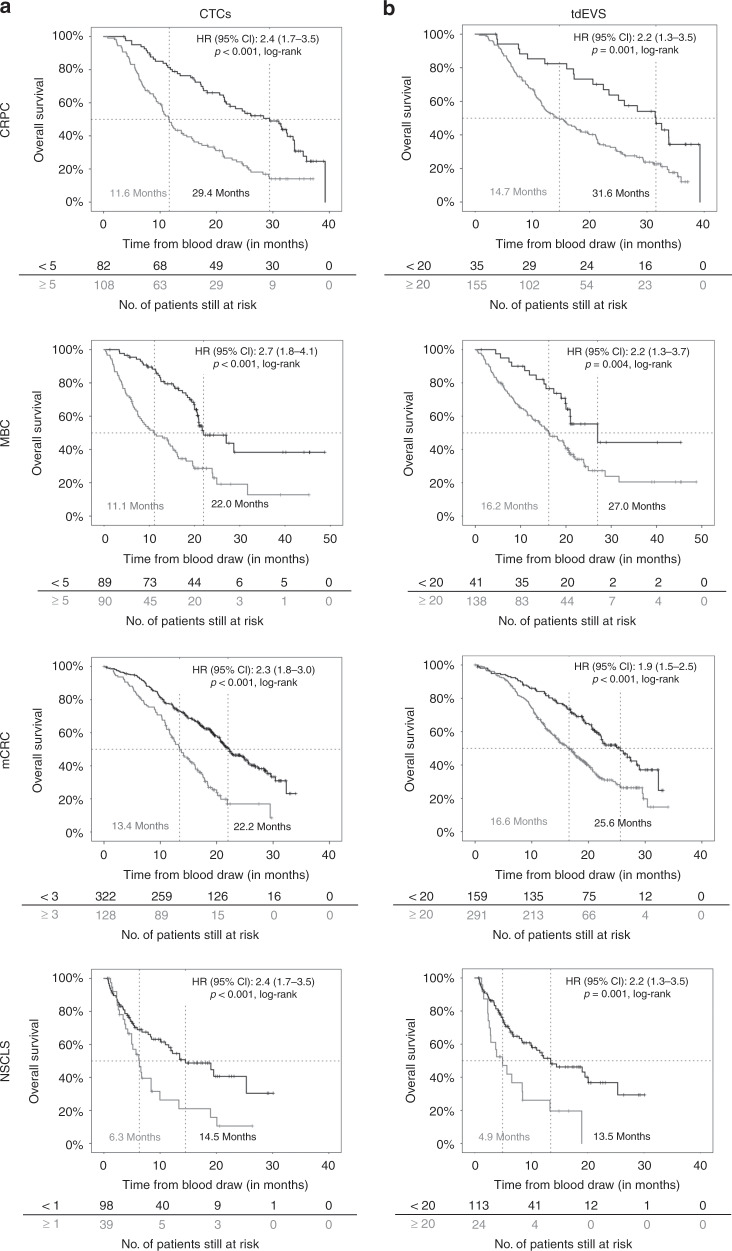
Kaplan–Meier plots for overall survival in CRPC, MBC, mCRC and NSCLC patients with favourable and unfavourable CTC (**a**) and tdEV (**b**) counts using different cut-off values for CTCs (5 for CRPC and MBC, 3 for mCRC and 1 for NSCLC) and the same cut-off value of 20 for tdEVs.

The association of CTCs and tdEVs in CRPC, MBC, mCRC and NSCLC with OS was evaluated using KM plots (Fig. 3). For CTCs, the same cut-offs established in the original studies (5 for CRPC and MBC and 3 for mCRC) were used to dichotomise patients into favourable and unfavourable CTC groups. For NSCLC patients, 1 CTC was used as a cut-off. For tdEVs, the cut-off value used to dichotomise patients into groups with favourable and unfavourable tdEVs was defined as the upper bound of the normal reference range (≥20 tdEVs). Univariable Cox proportional hazards regression analysis was used to estimate the HR for OS between the favourable and unfavourable CTC and tdEV groups. As shown in Fig. 3, cancer patients with ≥20 tdEVs/7.5 mL have an approximately twofold higher risk of death compared to patients with <20 tdEVs (*p* < 0.05, log-rank test). The stratification of the same patients based on their CTC counts resulted in slightly higher HRs for OS in all cancer types, except for NSCLC, where the HR was slightly higher for tdEVs (*p* < 0.05, log-rank test, Fig. 3).

**Fig. 4:**
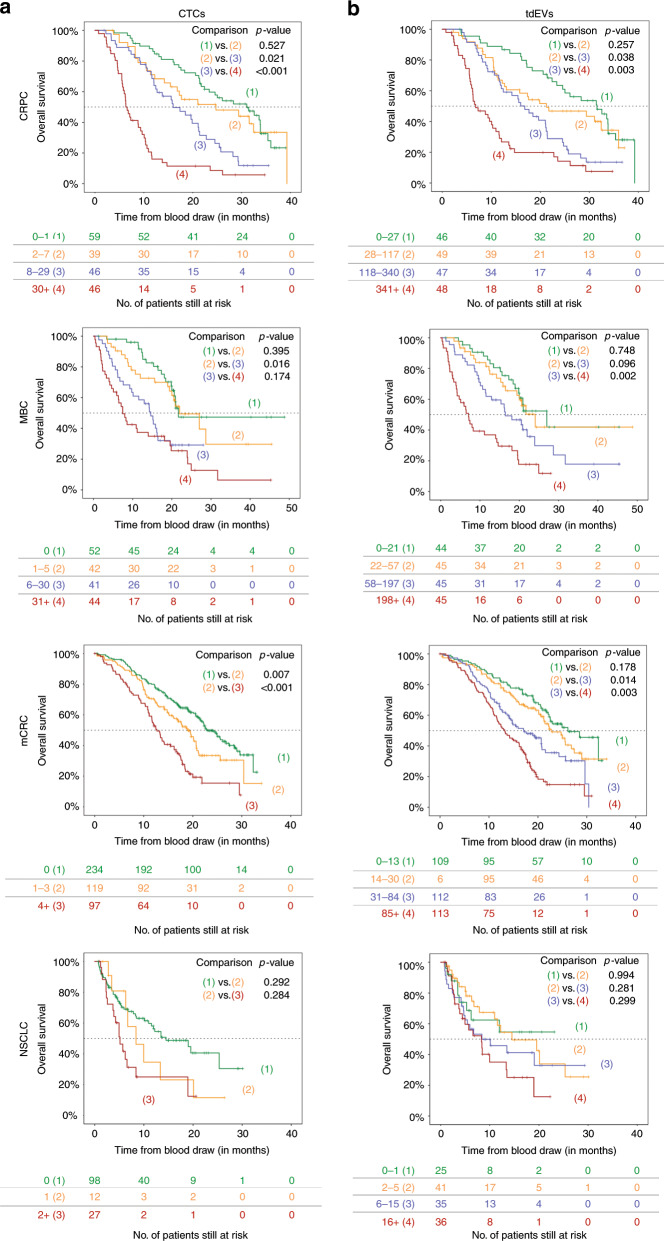
Kaplan–Meier plots for overall survival in CRPC, MBC, mCRC and NSCLC patients with increasing levels of CTCs (**a**) and tdEVs (**b**). Increasing tdEV counts are associated with worse OS in all cancer types, similarly to CTCs.

To demonstrate the association between increasing CTC and tdEV load and OS, KM plots were generated. For this analysis, CTCs and tdEVs for each cancer type were binned into four groups, except for the CTC analysis of mCRC and NSCLC, where the CTC counts were binned into only three groups because the majority of patients did not have CTCs. As shown in Fig. 4, increasing tdEV load in the blood of cancer patients (Fig. 4b) is significantly associated with worsening OS. The same pattern can be seen for CTCs (Fig. 4a), although the narrower range of CTC values leads to more unequal numbers of patients in each risk group compared to their stratification based on tdEV values.

To investigate the dependence of HR for all four different cancer types on different cut-off values for both CTCs and tdEVs, overview plots of HR (including 95% CIs) for OS were generated by the open-source Cutoff Finder web application (Supplementary Fig. S2). For CRPC, MBC and mCRC cancer, >94% of all possible cut-off values for both CTCs and tdEVs lead to a significant dichotomisation of patients for OS. For NSCLC, all of the possible CTC cut-off values and 67% of possible tdEV thresholds resulted in patient stratification with a significant HR. Based on the same overview plots, it is clear that HRs for both CTCs and tdEVs in all different cancer types are similar.

The overlap of ROC curves that were generated to evaluate the performance of CTCs and tdEVs in classifying patients based on their OS and experiencing death (Supplementary Fig. S3) further supports the finding that CTCs and tdEVs are equivalent prognostic biomarkers of OS.

### Stratification of patients with favourable CTC counts based on tdEVs

**Fig. 5:**
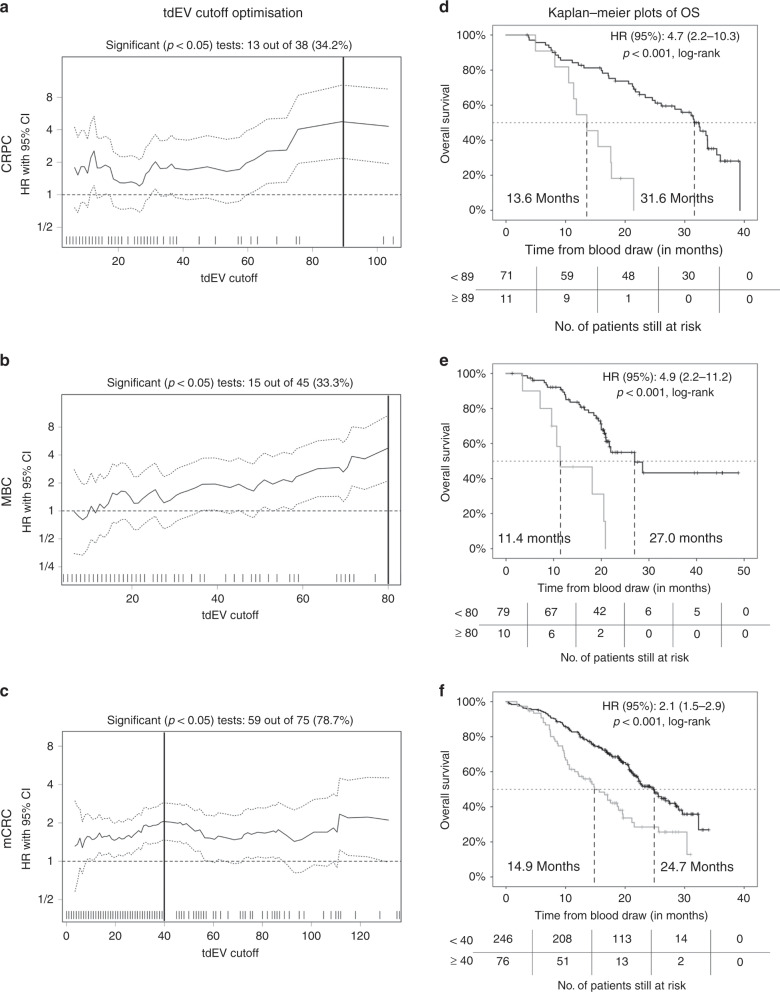
Overview plots of HRs (with 95% CIs) for all possible cut-off values for tdEV counts in CRPC (**a**), MBC (**b**) and mCRC (**c**) patients with favourable CTC counts (<5 for CRPC and MBC and <3 for mCRC), as generated by the Cutoff Finder software. The optimal cut-offs, determined as the point that resulted in the most significant split (log-rank test), are indicated by a vertical line in each plot. Kaplan–Meier plots for overall survival in CRPC (**d**), MBC (**e**) and mCRC (**f**) patients with favourable CTC counts by favourable and unfavourable tdEV counts were generated based on the optimal tdEV cut-off values. Favourable CTC patients with unfavourable (elevated) tdEV counts had significantly worse OS compared to favourable CTC patients with favourable (below the selected cut-off values) tdEV counts.

To evaluate whether patients with favourable CTC counts could be further stratified using tdEVs, the Cutoff Finder web application was used to plot the resulting HRs (with 95% CI) against the different cut-off values of tdEVs for each cancer type (Fig. 5a–c). For CRPC, the optimal tdEV cut-off (i.e. the cut-off leading to the highest HR with a *p* value <0.001, log-rank test) was ≥89, with 13% of the patients with favourable CTC counts having elevated (unfavourable) tdEV counts. For MBC, the optimal tdEV cut-off was ≥80, with 11% of the patients with favourable CTC counts having elevated tdEVs. For mCRC, the optimal tdEV cut-off was ≥40, with 24% of the patients with favourable CTC counts having unfavourable tdEV counts. For NSCLC, no cut-off value for tdEVs was found that led to a significant dichotomisation of patients with favourable CTC counts. KM plots were generated for patients with favourable CTC counts based on the aforementioned optimal tdEV cut-off values for each cancer (Fig. 5d–f). Patients with unfavourable tdEV counts had significantly worse OS in comparison to patients with favourable tdEV counts, with a 4.7-fold higher risk of death (95% CI: 2.2–10.3) in CRPC, a 4.9-fold higher risk (95% CI: 2.2–11.2) in MBC and a 2-fold higher risk (95% CI: 1.5–2.9) in mCRC.

### Univariable and multivariable associations between potential risk factors and OS of CRPC, MBC, mCRC and NSCLC patients

Different variables for each cancer type were evaluated as potential risk factors of OS using univariable Cox proportional hazards regression analysis (Supplementary Table S2). For the multivariable analysis, the significant predictors of the univariable analysis were included (Supplementary Table S3).

In case of CRPC, the tested variables were age, Eastern Cooperative Oncology Group (ECOG) status, haemoglobin and the log-transformed CTCs, tdEVs, prostate-specific antigen, lactate dehydrogenase (LDH), alkaline phosphatase, albumin and testosterone. All variables, except for testosterone, were significant and used as input variables in the multivariable regression analysis. The final multivariable model included ECOG status, age, haemoglobin, tdEVs and LDH.

In case of MBC, ECOG status, age, oestrogen receptor, HER2 and progesterone receptor (PR) status of the tumour, time to metastasis, number of metastatic sites, line of therapy, type of therapy and log-transformed CTCs and tdEVs were evaluated as potential risk factors. ECOG status, PR status, number of metastatic sites, type of therapy, CTCs and tdEVs were significant and used as input variables in the multivariable regression analysis. The final multivariable model included the number of metastatic sites, ECOG status and CTCs.

In case of mCRC, the tested variables were the treatment arm, ECOG status, age, LDH (normal versus abnormal), gender, prior adjuvant therapy, >1 affected organs and the log-transformed CTCs and tdEVs. All variables apart from the treatment arm, the prior adjuvant therapy and the gender were significant predictors of OS and were used as input variables in the multivariable regression analysis. The final multivariable model included the ECOG status, age and both CTCs and tdEVs.

In case of NSCLC, the tested variables were the ECOG status, type of treatment, age, gender and the log-transformed CTCs and tdEVs. Except for the type of treatment, all variables were significant predictors of OS and used as input variables in the multivariable regression analysis. The final multivariable model included the ECOG status, age and CTCs.

## Discussion

The FDA-cleared CellSearch® system presents thumbnail images containing CK-PE and DAPI signals to the operator for manual classification of CTCs.^39^ The operator bias in CTC classification can be improved by providing quantitative information of the objects in the thumbnails and can be eliminated by the use of gates with the open-source ACCEPT software (http://github.com/LeonieZ/ACCEPT).^14,41–43^ Further improvements in the classification of CTCs can be obtained through the use of deep learning;^44^ however, for its implementation on larger data sets, significant improvements in the software are still needed. Therefore, in this study, we elected to use the original CTC scores of the studies. The introduced operator bias together with the assay variations and the low cut-off values of 1, 3 and 5 CTCs per 7.5 mL of blood make an accurate assessment impossible. As an example, two separate tubes of blood drawn from the same patient at the same time could very likely show that one tube had four CTCs, while the other tube had six CTCs, resulting in the assignment of the patient into a different risk group.^45^

For tdEVs, no manual scores can be obtained as the CellSearch generated thumbnails do not include objects with only CK-PE. Whether or not deep learning can be also used to improve detection and classification of tdEVs remains, as of yet, unanswered. Notably, ACCEPT tdEV counts are found in at least one order of magnitude higher frequencies compared to CTCs in all different cancer types, thereby confirming our previous findings in CRPC.^14^ The high degree of correlation between CTCs with tdEVs that we found in CRPC, MBC and mCRC (Supplementary Fig. S1) can likely be attributed to a similar shedding of tdEVs from the primary and metastatic sites as with the CTCs. Origin of tdEVs by degradation of CTCs after blood draw is unlikely, as the blood samples were collected in CellSave tubes, which stabilises the cells in the blood, preventing their degradation.^46^

One way for the clinicians to bring our findings into patient care is by automatically extracting tdEV counts of the already processed samples for CTCs; the tdEV count can serve as a second biomarker to confirm the initial prognosis of the patient based on their CTC counts. However, for the additional stratification of patients with low (favourable) CTC counts reported here, the tdEV cut-off values with the most significant correlation to OS were chosen for each patient cohort. It is recommended that the selected tdEV cut-off values be confirmed in additional studies to derive more unequivocal conclusions.

In case of NSCLC patients, tdEVs are unable to further stratify patients with favourable CTC counts. Moreover, both CTC and tdEV counts, as defined by the CellSearch system, are very low regardless the particular aggressiveness of the specific cancer type. The question why remains open and has been discussed elsewhere.^47^ Low expression of EpCAM and/or CK has been suggested as possible explanations. The CellSearch yields a CTC recovery of 87–91% when cells express 1.5–2.3 × 10^6^ EpCAM molecules.^35^ However, when EpCAM expression falls to 4.9–2.0 × 10^3^ EpCAM molecules, the respective CTC recovery drops to as low as 2%.^35^ In case of the smaller tdEVs, lower magnetic forces and subsequently EpCAM expression is required for their immunomagnetic isolation. Another critical point is the detection of isolated EpCAM+ CTCs by the expression of CK8, 18 and 19. Colleagues have previously demonstrated that androgen receptor-positive (AR+) CTCs isolated by the CellSearch system were not detectable by the CellTracks Analyzer II due to insufficient CK expression.^48^ de Wit et al. also reported that, in case of NSCLC patients, the inclusion of antibodies targeting more CKs increased the detection of CTCs within the CellSearch cartridges from 41% to 52%.^35^ Therefore, the use of additional antibodies recognising membrane (EpCAM, VAR2CSA, HsP70, PSMA, HER2, epidermal growth factor receptor 2 (EGFR2)) or cytoplasmic (vimentin) markers could lead to increased detection of CTCs and tdEVs that have been already positively selected based on their EpCAM expression.

The inclusion of more specific antibodies in the staining mixture could also aid in the discrimination of tdEVs from EVs secreted from non-tumour epithelial cells in the bloodstream of healthy individuals. The reason why only some of the healthy individuals have detectable EVs defined as tdEVs (Fig. 2) could be explained by a condition of liver inflammation/fibrosis that would result in increased secretion of epithelial EVs. In alignment with that hypothesis, Julich-Haertel et al. demonstrated that individuals with cirrhosis have detectable EpCAM+ AnnexinV+ EVs in similar frequencies to individuals with NSCLC and CRC.^49^

Genotypic characterisation of tdEVs in healthy individuals and cancer patients would further confirm their origin. Molecular profiling of the isolated tdEVs could provide us with a mutational snapshot of the tumour during therapies and lead to a better understanding of the underlying pathways and mechanisms that promote progression. Jiang et al. identified AR mutations in CellSearch-enriched blood samples of CRPC patients that did not have any CellSearch-defined CTCs; some of the AR mutations that were indicating resistance to AR-targeted therapies had been found also in biopsies or autopsies of the respective patients.^50^ Furthermore, Marchetti et al. also reported EGFR mutations in CellSearch-enriched blood samples of 84% NSCLC patients, whereas only 41% of them were positive for CellSearch-defined CTCs.^51^ These findings raise the question whether specific gene mutations are present in EpCAM+ CTCs isolated by CellSearch but missed by the system as CK− or whether these mutations are encapsulated within the EpCAM+, CK+, CD45-, DNA- tdEVs that we report here. Both hypotheses are likely to be true as supported by the presence of CK− CTCs in the CellSearch cartridges^48^ and the presence of most tumour DNA in large CK+ tdEVs found in the plasma of cancer patients.^10^ In addition, it has been reported that relevant gene mutation information is present in the plasma fraction of blood samples, either encapsulated within EVs or as cell-free tumour DNA.^52,53^

Our study has the limitation that all blood samples were centrifuged at 800 × *g* for 10 min and the plasma was aspirated before being processed by the CellSearch system; hence, the EpCAM+, CK+, DAPI−, CD45− fraction of tdEVs that we report here was isolated from the blood cell pellet and it has a size range between 1 and 14 μm. These large tdEVs constitute only a small subset of probably <1% of the total tdEVs that are present in the blood sample before centrifugation, based on the size estimations of secreted EVs from model cancer cell lines.^54–56^

An approach to enrich and further investigate the smaller tdEVs is to run plasma through the CellSearch system. A first attempt indeed showed many CK+ tdEVs isolated from the plasma of a CRPC patient by the CellSearch system, but improvements need to be made for their detection and identification. Further characterisation and downstream analysis of the EpCAM-enriched tdEVs using gold standard techniques,^57,58^ also recommended by the International Society of Extracellular Vesicles,^59^ can further elucidate their biophysical properties. Towards that direction, the Cancer-ID consortium was formed to identify and characterise tdEVs and elucidate their differences from EVs of different origins. Electron microscopy,^60,61^ nanoparticle tracking analysis, flow cytometry,^62,63^ Raman spectroscopy,^64^ surface plasmon resonance^55^ and atomic force microscopy^65^ have been investigated for the characterisation of tdEVs with or without EpCAM pre-enrichment. Principal component analysis of Raman spectra shows clear discrimination between EVs of cancerous cell and healthy blood cell origin.^54^

In conclusion, this study shows the simultaneous isolation and detection of CTCs and large (1–14 μm) tdEVs in a single assay (CellSearch) maximising the available data from individual peripheral blood samples of metastatic cancer patients. Importantly, tdEVs have an equivalent prognostic power to CTCs in CRPC, MBC, mCRC and NSCLC patients and can further stratify patients with low/favourable CTC counts. Furthermore, the non-stringent criteria used to classify an object as a tdEV allows for their reliable and fast automated enumeration using the ACCEPT software without the necessity of time-consuming training and manual scoring by individual users. The presence of tdEVs in higher frequencies, when compared to CTCs, may better reflect the phenotypic heterogeneity of the tumour. That fact together with the increased stability of EVs in circulation render tdEVs as a promising biomarker for clinicians to evaluate the mutational status, transcriptome and proteome of the tumour and the presence of therapeutic targets that could predict treatment responses of patient subsets with or without CTCs. Last but not least, future research of EVs could contribute in the comprehension of the underlying mechanisms of the tumour to develop resistance to treatments and open the path towards the development of new therapies.

## Supplementary information

Supplementary data

## Data Availability

The data sets used and analysed during the current study are available from the corresponding author on reasonable request.
