# Supplementary material for: Tumour-derived extracellular vesicles in blood of metastatic cancer patients associate with overall survival
Source: Br J Cancer. 2020 Jan 15;122(6):801–11. doi: 10.1038/s41416-019-0726-9 (PMC7078322; doi:10.1038/s41416-019-0726-9)
Supplement: Supplementary file 1 — Supplementary data [file 41416_2019_726_MOESM1_ESM.docx]

***Supplementary Figure S1****: Scatter plot showing the correlation between manual CTC and automated tdEV counts in four cancer types and healthy donors. Each group is indicated in a different color as shown in the legend.*


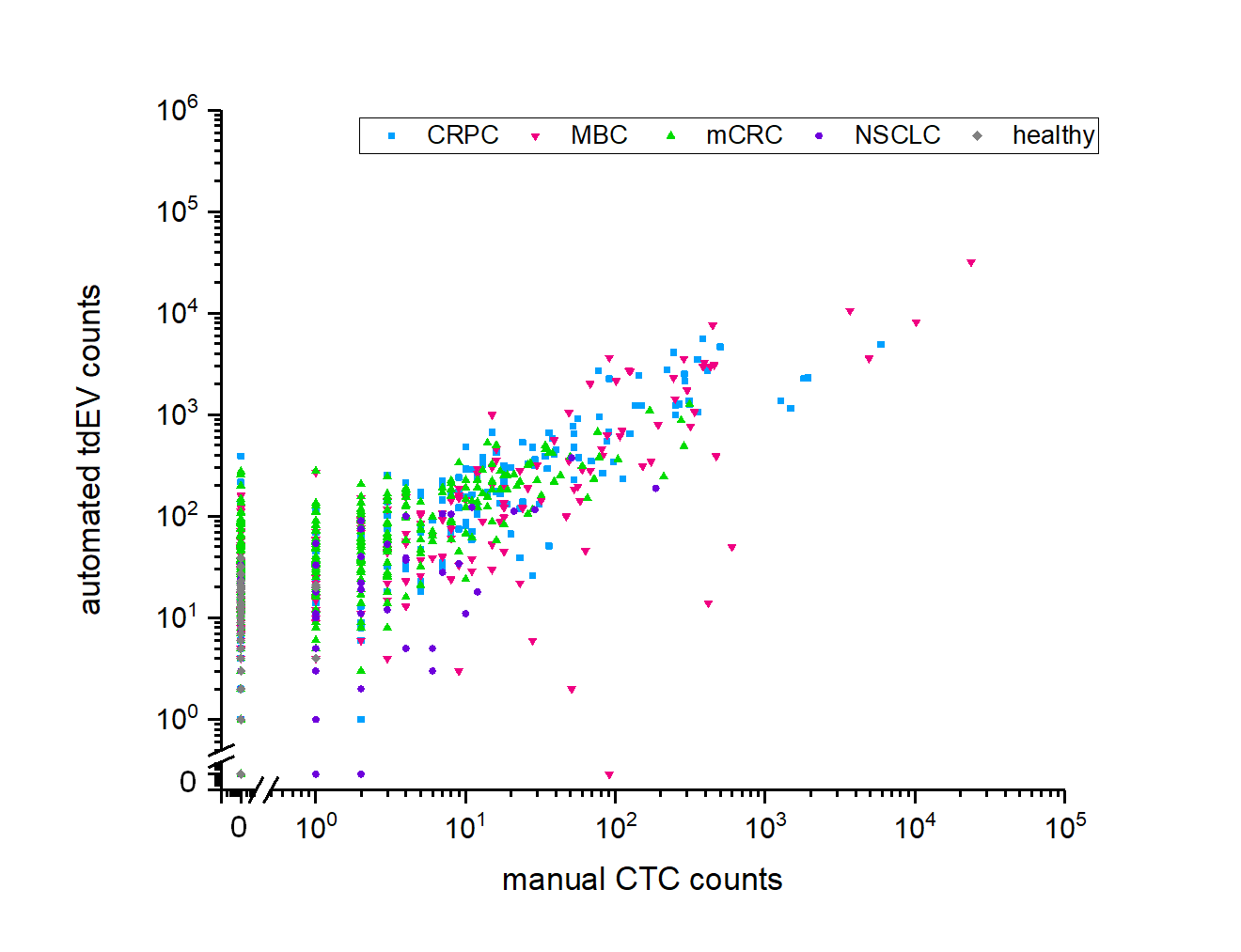


***Supplementary Figure S2****: Overview plots of Hazard Ratios (HRs), including 95% CIs, for overall survival (OS) for all possible cut-off values for CTC counts (A) and tdEV counts (B) in each cancer type. These plots were generated using the web application Cutoff Finder. CTCs and tdEVs were significant for a wide range of cut-off values in CRPC (Panels 1A and 1B), MBC (Panels 2A and 2B), mCRC (Panels 3A and 3B), and NSCLC (Panels 4A and 4B).*

**
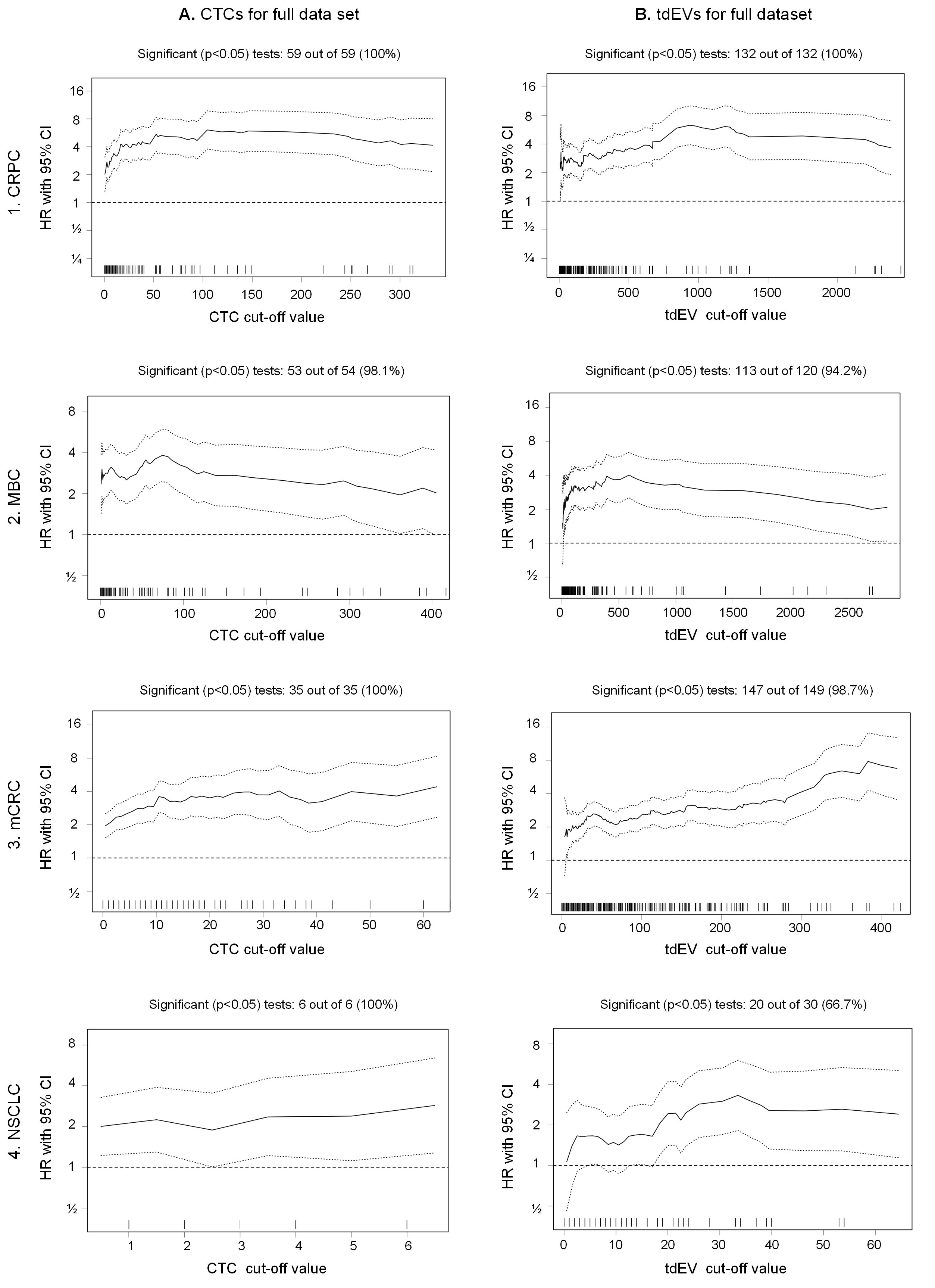
**

***Supplementary Figure S3*:** *Receiver Operating Characteristic* *(ROC) curves of CTCs (in black) and tdEVs (in grey) treating as a classification variable A. survival time dichotomized by the median OS time of the respective patient cohort and B. death.*

***
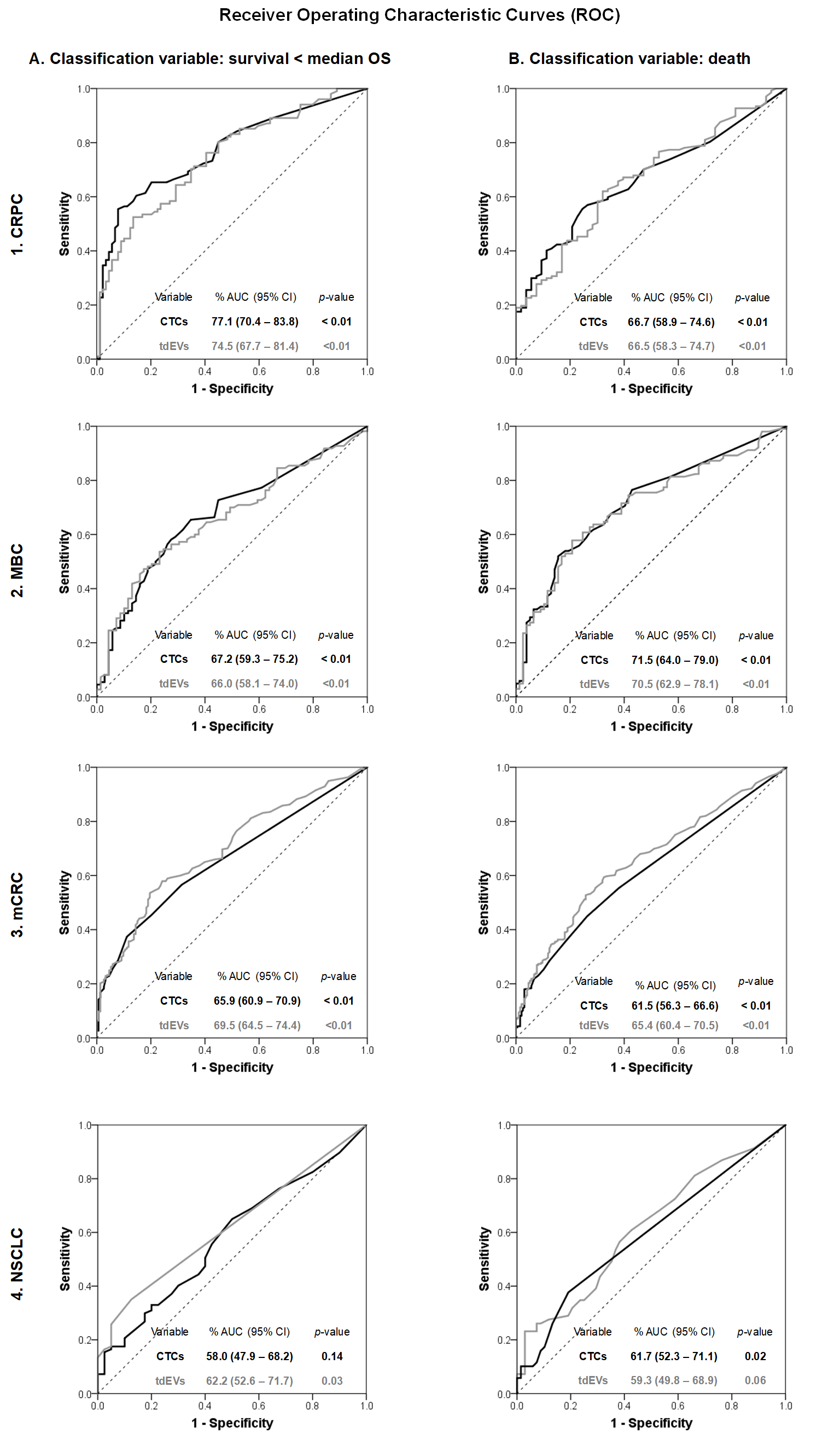
***

**Supplementary Table S1: 137 NSCLC patient samples included in the present study**

| N patients | Additional labeling | Additional staining | Previously reported |
| --- | --- | --- | --- |
| 45 | Marker 1  Marker 2 | Wheat-germ agglutinin- Alexa 488  CD16-PerCP | (de Wit et al., 2018b) |
| 12 | Marker 2 | CD16-PerCP | not |
| 24 | Marker 1  Marker 2 | CK1-8, 10, 14, 15, 16, 19, 20-FITC  CD16-PerCP | (de Wit et al., 2015) |
| 56 | Marker 2 | CD16-PerCP | (de Wit et al., 2018a) |

**Supplementary Table S2: Univariable cox proportional hazards regression analyses**

|  | Variables in equation | HR (95% CI) | *p*-value |
| --- | --- | --- | --- |
| CRPC | age | 1.02 (1.00-1.04) | 0.025 |
|  | ECOG | 2.33 (1.75 - 3.10) | <0.001 |
|  | CTCs, per log unit | 1.72 (1.48 - 2.00) | <0.001 |
|  | tdEVs, per log unit | 2.42 (1.90-3.07) | <0.001 |
|  | haemoglobin, per g/dL | 0.69 (0.62 - 0.77) | <0.001 |
|  | PSA, per log unit | 1.27 (1.00- 1.62) | 0.050 |
|  | ALP, per log unit | 3.70 (2.31 - 5.93) | <0.001 |
|  | albumin, per log unit | 0.01 (0.00 - 0.02) | 0.002 |
|  | testosterone, per log unit | 0.71 (0.35 - 1.42) | 0.328 |
|  | LDH, per log unit | 26.54 (11.28 - 62.48) | <0.001 |
| MBC | age | 1.00 (0.98 - 1.01) | 0.526 |
|  | ECOG | 1.81 (1.39 - 2.36) | <0.001 |
|  | CTCs, per log unit | 1.62 (1.38 - 1.90) | <0.001 |
|  | tdEVs, per log unit | 1.92 (1.53 - 2.40) | <0.001 |
|  | ER status | 1.48 (0.97 - 2.24) | 0.068 |
|  | PR status | 1.57 (1.06 - 2.34) | 0.025 |
|  | HER2/neu status | 1.43 (0.89 - 2.30) | 0.136 |
|  | number of metastatic sites | 1.24 (1.07 - 1.43) | 0.003 |
|  | time to metastasis | 0.97 (0.93 - 1.00) | 0.067 |
|  | line of therapy | 1.06 (1.00 - 1.12) | 0.052 |
|  | type of therapy | 0.81 (0.67 - 0.97) | 0.021 |
| mCRC | age | 1.01 (1.00 - 1.03) | 0.039 |
|  | ECOG | 1.51 (1.18 - 1.92) | 0.001 |
|  | gender | 0.99 (0.77 - 1.27) | 0.936 |
|  | treatment arm | 1.14 (0.90 - 1.46) | 0.284 |
|  | prior adjuvant chemotherapy | 1.34 (0.93 - 1.93) | 0.117 |
|  | > 1 affected organs | 0.76 (0.59 - 0.98) | 0.035 |
|  | abnormal LDH | 0.59 (0.46 - 0.75) | <0.001 |
|  | CTCs, per log unit | 1.65 (1.44 - 1.90) | <0.001 |
|  | tdEVs, per log unit | 2.53 (1.99 - 3.20) | <0.001 |
| NSCLC | age | 1.03 (1.00 - 1.06) | 0.024 |
|  | ECOG | 2.04 (1.36 - 3.04) | 0.001 |
|  | gender | 0.59 (0.36 - 0.95) | 0.030 |
|  | type of therapy | 0.98 (0.74 - 1.31) | 0.907 |
|  | CTCs, per log unit | 1.66 (1.24 - 2.23) | 0.001 |
|  | tdEVs, per log unit | 1.52 (1.06 - 2.19) | 0.024 |

**Supplementary Table S3: Multivariable cox proportional hazards regression analyses**

|  | Variables in equation | HR (95% CI) | *p*-value |
| --- | --- | --- | --- |
| CRPC | LDH, per log unit | 7.01 (2.47 - 19.96) | <0.001 |
|  | tdEVs, per log unit | 1.87 (1.42 - 2.47) | <0.001 |
|  | ECOG status | 1.62 (1.19 - 2.20) | 0.002 |
|  | haemoglobin, per g/dL | 0.80 (0.70 - 0.90) | <0.001 |
|  | age | 1.03 (1.01 - 1.05) | 0.003 |
| MBC | CTCs, per log unit | 1.62 (1.37 - 1.92) | <0.001 |
|  | ECOG status | 1.76 (1.34 - 2.30) | <0.001 |
|  | number of metastatic sites | 1.22 (1.05 - 1.41) | 0.008 |
| mCRC | tdEVs, per log unit | 1.92 (1.37 - 2.68) | <0.001 |
|  | ECOG status | 1.36 (1.06 - 1.74) | 0.016 |
|  | CTCs, per log unit | 1.23 (1.01 - 1.49) | 0.041 |
|  | age | 1.02 (1.01 - 1.04) | 0.005 |
| NSCLC | ECOG status | 1.90 (1.26 - 2.86) | 0.002 |
|  | CTCs, per log unit | 1.47 (1.05 - 2.07) | 0.026 |
|  | age | 1.04 (1.01 - 1.06) | 0.010 |
